# Effects of different pioneer and exotic species on the changes of degraded soils

**DOI:** 10.1038/s41598-022-23265-y

**Published:** 2022-11-03

**Authors:** Claudio Cacace, Juan C. García-Gil, Claudio Cocozza, Francesco De Mastro, Gennaro Brunetti, Andreina Traversa

**Affiliations:** 1 Dipartimento di Scienze del Suolo, della Pianta e degli Alimenti, University of Bari, 70126 Bari, Italy; 2 Instituto de Ciencias Agrarias, Consejo Superior de Investigaciones Científicas (CSIC), Serrano 115 bis, 28006 Madrid, Spain

**Keywords:** Carbon cycle, Enzymes, Forestry

## Abstract

Soil degradation resulting from deforestation contributes to a dramatic decline in soil quality whose restoration must go through reforestation with pioneer species. We investigated the effects of cypress and black locust, pioneer but exotic species, on soil chemical properties and microbial and enzymatic activities of two marginal soils. The sampling sites were Lama Giulia and Locone lake in the Murge plateau of the Apulia Region, Italy. The soils at Lama Giulia presented a silty loam texture, while at Locone Lake site were sandy, and most likely due to the different texture, the former exhibited higher organic C, N, P and micronutrients contents than Locone Lake under black locust reforestation, despite the latter was reforested earlier. In addition, the higher microbial entropy and turnover of Locone Lake’s soils suggested a less conservative soil state than Lama Giulia’s soils. The effects of black locust reforestation at Lama Giulia on almost all soil parameters considered did not differ from those of the corresponding pasture, confirming the more conservative soil state in that site and suggesting that the time of reforestation was not enough to get differences between the reforested and not reforested soil. The soils reforested with cypress showed the significantly highest SOC, N, dissolved organic C and microbial biomass C content. In addition, it presented also the numerically largest dehydrogenase, phosphatase and β-glucosidase activities, soluble carbohydrates, and phenolic compounds content. These results may be ascribed to the longer litter deposition occurred in cypress soils.

## Introduction

Land degradation is caused by natural and anthropogenic processes threatening the capacity of natural resources to carry out their ecological functions^1^. Soil degradation is related to a decline in the multiple ecosystem services relying on it, as a result of adverse changes in its biological, chemical, physical and hydrological properties^2^, affecting the soil capacity to perform a range of ecological functions^3^.

**Figure 1:**
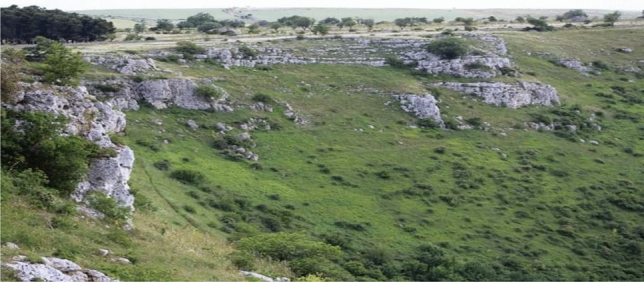
Typical landscape of Murge plateau.

In the north-western sectors of Murge plateau, an inland part of Apulia region, southern Italy (Fig. 1), land degradation is mainly attributed to the intensive deforestation that started since the roman time due to the large amount of timber removed, which was necessary for the construction of warships^4^. The original floristic composition of the north-western sectors of Murge plateau was dominated by *Quercus* *dalechampii* Ten. and *Quercus* *virgiliana* Ten., with the contribution of species belonging to the *Quercetalia* *pubescenti—petraeae* order. The *Stipo* *Bromoidis—Quercetum* *Dalechampii* phytocoenosis was completed by *Stipa* *bromoides* L.*,* *Crataegus* *laevigata* (Poir.) DC., *Lonicera* *etrusca* Santi*,* *Carex* *hallerana* Asso*,* and *Iris* *collina* N. Terracc. The ancient forest is today reduced to remnant strips because of the intense exploitation for coppicing, and sheep and goat pasturing^5^. The pastures not protected by trees were affected by intense erosion and “evolved” into degraded pastures, shallow soils or even outcropping rocks^6^.

In order to solve such critical situation, several reforestations were realized over time utilizing pioneer species, such as *Pinus* *halepensis* L., *Pinus* *pinea* L. and *Cupressus* *spp.,* and the nitrogen fixing black locust (*Robinia* *pseudoacacia* L.), due to their ability to grow up on shallow and eroded soils. Although the black locust does not enjoy a good reputation as a forest tree because of its invasiveness^7,8^, it has been utilized extensively for reforestation. These pioneer trees prevent erosion by covering the soil, reduce the impact of rainstorms erosion and produce, over time, litter and root exudates that increase the depth and the organic matter content of soils and can modify the biogeochemical cycles of N and C. These circumstances restore the soil ecological functions to reestablish native forestry species, such as several oaks, walnut, linden, etc., that need higher level of soil quality in comparison to pioneer forestry species.

Since the reforestations have not been conducted in a homogeneous way, in the Murge plateau one can find reforestations that differ in age of plantation and pioneer trees used. Therefore, we selected two sites that diverge for the aforementioned characteristics and investigated their effects on chemical, microbial, and enzymatic soil properties involved in the biogeochemical nutrients cycling, such as microbial biomass, organic matter, soil enzyme activities and soil microbial entropy that are some of the most well-known soil quality indicators^9,10^.

## Materials and methods

### Site description and samples collection

The Murgia plateau decreases its elevation from about 680 m a.s.l down to the Adriatic Sea, is characterized by an annual rainfall of about 650 mm^11^, concentrated mainly in fall and winter, and the rainwater reaches the sea through several incised karst valleys, locally called “lame”, that act as ephemeral streams only during the rainstorms^12^. From 2004 this area belongs to the “Alta Murgia National Park”.

The first sampling site was Lama Giulia (40°57′12.21′′ N; 16°20′14.75′′ E), belonging to the municipality of Gravina in Puglia, characterized by Calcic Haploxeralf fine loamy mixed thermic soils^13^. It was reforested with *Cupressus* *arizonica* Greene (CYG) during the years 1976–1980 (about 40 years of reforestation at the time of soil sampling), and with *Robinia* *pseudoacacia* L*.* (ROG), whose plantation dates to the end of 1990’s—beginning of 2000’s (about 17 years of reforestation at the time of soil sampling)^14^. *Cupressus* *arizonica* Greene is a conifer belonging to the family of Cupressaceae native to Arizona and New Mexico. It is also used as an ornamental tree, for timber and as a windbreak^15^. *Robinia* *pseudoacacia* L. is a broad-leaf tree belonging to the family of Leguminosae native to the North America. It is a promising fast-growing species for biomass production in short rotation coppice system^16^. It is also used as ornamental tree and windbreak, and for honey production^8^. The pasture near CYG and ROG, consisting in degraded but not reforested fields, was considered as control (COG) to check the effects of reforestation. It is mainly composed of shrubs and herbs belonging to four vegetation classes such as *Artemisietea* *Vulgaris*, *Lygeo-Stipetea*, *Festuco-Brometea*, and *Stellarietea* *Mediae*^17^. This site is close to an abandoned enclosure built with dry stone walls, commonly used in the past for sheltering sheeps and goats.

The other sampling site was located near the Locone Lake (41°04′52.88′′ N; 16°00′54.45′′ E), belonging to the municipality of Minervino Murge, characterized by Typic Calcixerept sandy mixed thermic soils^13^. It was mainly reforested with *Robinia* *pseudoacacia* L*.* (ROM) in 1985 (about 30 years of reforestation at the time of soil sampling)^18^. As per Lama Giulia, the pasture near ROM was considered as control (COM). It is currently characterized by arid mediterranean meadows comprising *Brachipodium* *resutum* (Pers.) P.Beauv., *Brachipodium* *ramosum* Roem. & Schult., *Trachynia* *distachya* (L.) P.Beauv., *Bromus* *madritensis* L., and *Lagurus* *ovatus* L.^19^. This site shows no signs of farming, even in the past, so grazing is presumably due only to wild herbivores.

Each site was sampled in spring 2017 using an auger, removing the litter and the herbaceous layer, and coring the soil until the abundant root apparatus depth (about 20–25 cm). In details, three composite samples (each made of five subsamples) were cored from CYG, ROG, COG, ROM, and COM, air dried, gently crushed and passed through a 2-mm sieve.

### Physical and chemical soil analyses

Soil samples were analyzed according to standard methods^20^. The soil organic carbon (SOC) content was measured by the Walkley–Black method, and the SOC enrichment was calculated as the difference between the reforested SOC content and the SOC content of the corresponding control soil (pasture). The total nitrogen (TKN) was determined by the Kjeldahl method and the TKN enrichment was calculated as per SOC enrichment. The available phosphorus (P_ava_) was determined by UV–Vis spectrophotometry according to Olsen method. The exchangeable Ca and K were extracted with a BaCl_2_ and triethanolamine solution buffered at pH 8.2 and determined using the inductively coupled plasma optical emission spectrometer (ICP-OES) iCAP 6300 (Thermo Electron). The available fractions of Fe, Zn, Cu, Mn and Mo were extracted with diethylenetriaminepentaacetic acid (DTPA) and quantified using the ICP-OES.

Particle-size distribution was determined by the pipette method, and the soil texture was identified using the USDA soil textural classification system^13^.

Dissolved organic carbon (DOC), soluble carbohydrates and phenolic compounds of soil were analyzed on water extracts obtained at a soil to water ratio 1:10. DOC was determined with a Shimadzu TOC-VCSH analyzer (Kyoto, Japan). Soluble carbohydrates were determined spectrophotometrically by the anthrone method^21^ and expressed as glucose C equivalents (mg C kg^-1^). Phenolic compounds were determined by the method of Lowry et al.^22^ based on the Folin-Ciocalteu reagent and expressed as p-coumaric acid equivalents (mg kg^-1^).

### Soil biochemical analyses

Dehydrogenase activity was measured as the reduction of 2-*p*-iodophenyl-3-*p*-nitrophenyl-5-phenyltetrazolium chloride (INT) to iodonitrophenyl-formazan (INTF)^23^ and expressed as micrograms of INTF produced per soil gram (dry mass) per h (μg INTF g^−1^ h^−1^). Microbial biomass C (MBC) content was determined by fumigation of the soil sample with ethanol-free CHCl_3_ and extraction with 0.5 M K2SO4, according to the method of Vance et al.^24^ modified by Gregorich et al.^25^, measured in a Shimadzu TOC-VCSH analyzer (Kyoto, Japan) and expressed in mg C per soil kg.

Alkaline phosphatase activity was determined following the method of Nannipieri et al.^26^, using *p*-nitrophenyl phosphate disodium (pNPP, 0.115 M) as substrate for this enzyme. This assay is based on the release and detection of *p*-nitrophenol (pNP) measured spectrophotometrically. β-glucosidase activity was analyzed according to the method of Tabatabai^27^, using p-nitrophenyl β-d-glucopyranoside (pNG, 0.05 M) as substrate for this enzyme. Phosphatase and β-glucosidase activities were expressed in micromoles of p-nitrophenol (pNP) produced per soil gram (dry weigh) per h (μmol pNP g^−1^ h^−1^).

Urease and protease activities were determined in 0.1 M phosphate buffer at pH 7; 1 M urea and 0.03 M *N*-α-benzoyl-argininamide (BAA) were used as substrates, respectively. Two mL of buffer and 0.5 mL of substrate were added to 0.5 g of the soil sample, which was incubated at 30 °C (urease) or 39 °C (protease) for 90 min. Both enzymatic activities were determined by the amount of NH_4_^+^ released^26^, expressed in units of micromoles of ammonium-N produced per soil gram (dry mass) per h (μmol NH_4_^+^-N g^−1^ h^−1^).

### Statistical analysis

All experimental data were tested against the normal distribution of variables (Shapiro–Wilk’s test) and the homogeneity of variance (Bartlett’s test) using the RStudio software. The variables normally distributed and with homogeneity of variances were subjected to one-way ANOVA and HSD test, while results not normally distributed were subjected to Levene’s test to check the homogeneity of variance: data with heterogeneous variance were subjected to the nonparametric Friedman test.

The principal component analysis (PCA) was performed to compare the two sites that had different chemical, microbial, enzymatic and botanical characteristics. It was achieved using XLSTAT software and shown as biplots of scores (sites) and loadings (variables).

The regression analyses were performed using the RStudio software and the goodness of fit was evaluated through R^2^.

## Results

All soils from Lama Giulia showed a silty loam texture, with an average composition of sand, silt and clay of 22.23, 54.04 and 23.73%, respectively, whereas all soils from Locone Lake were sandy (84% sand, 9.1% silt and 6.8% clay, on average).

**Figure 2:**
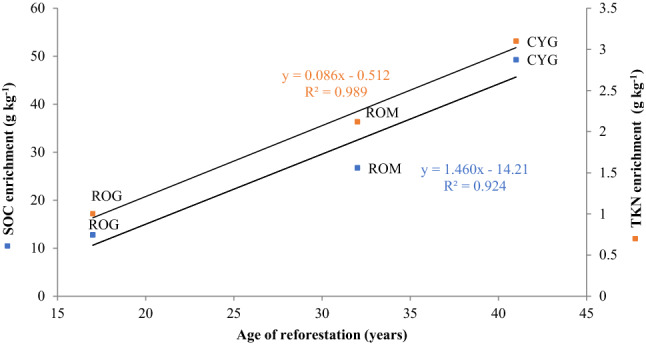
Linear regression between the age of each reforestation and the soil organic carbon enrichment. *ROG* black locust at Lama Giulia), *ROM* black locust at Locone Lake, *CYG* cypress at Lama Giulia.

Table 1 shows the average values of the main chemical parameters of the soil samples from Lama Giulia. Noteworthy, the SOC content was significantly (p = 0.0003) the highest in the soil samples of cypress with an average value of 94.3 g kg^−1^, followed by *Robinia* *pseudoacacia* L. (58.0 g kg^−1^) and pasture soils (45.1 g kg^−1^). The SOC enrichment of each reforested soil was linearly correlated with the age of each reforestation (Fig. 2). TKN resembled the SOC trend, with a significantly (p = 0.005) higher value recorded in soil samples from cypress (7.0 g kg^−1^) than black locust and pasture, that showed 4.9 and 3.9 g TKN kg^−1^, respectively. As per SOC, the linear correlation between the age of each reforestation and the TKN enrichment of each reforested soil showed that the differences of TKN content between the tree species was highly dependent by the time of planting (Fig. 2). In contrast, the P_ava_ was the significantly highest (p = 0.002) in the soil samples from black locust (390.33 mg kg^−1^), while in cypress and pasture soils was 14.1 and 32.66 mg kg^−1^, respectively. Finally, the black locust soil showed numerically and significantly higher content of exchangeable Zn and Cu (p = 0.03), respectively, than the other two soils.

**Table 1:** Chemical properties (mean ± standard deviation of three replicates) of Lama Giulia soils.

	SOC, g kg^−1^	TKN, g kg^−1^	P_ava_, mg kg^−1^	pH (H_2_O)	pH (KCl)	EC, μS cm^−1^	Ca_exc_, g kg^−1^	K_exc_, g kg^−1^	Fe_ava_, g kg^−1^	Zn_ava_, mg kg^−1^	Cu_ava_, mg kg^−1^	Mn_ava_, mg kg^−1^	Mo_ava_, mg kg^−1^
COG	45.1 ± 7.5 (b)	3.9 ± 0.8 (c)	32.66 ± 30.8 (b)	7.6 ± 0.2 (a)	6.8 ± 0.2	379 ± 67	6.05 ± 7.8	2.51 ± 0.5	3.70 ± 0.7	76.14 ± 12.3	39.02 ± 0.7 (ab)	941 ± 54	0.06 ± 0.07
CYG	94.3 ± 6.2 (a)	7.0 ± 0.5 (a)	14.06 ± 3.4 (b)	7.3 ± 0.1 (b)	6.7 ± 0.2	258 ± 75.3	3.01 ± 0.3	2.32 ± 0.2	3.73 ± 0.2	80.15 ± 3.2	38.19 ± 5.7 (b)	1146.6 ± 370	0.09 ± 0.05
ROG	58.0 ± 7.2 (b)	4.9 ± 0.8 (b)	390.33 ± 136.7 (a)	7.3 ± 0.1 (b)	6.6 ± 0.2	399 ± 22	5.26 ± 3.7	2.67 ± 0.3	3.29 ± 0.4	130.06 ± 23.8	53.03 ± 8.1 (a)	1235 ± 210	0.12 ± 0.01
	***	**	**	*	ns	ns	ns©	ns	ns	ns©	*	ns	ns

*COG* control soil, *CYG* cypress soil, *ROG* *Robinia* *pseudoacacia* L. Soil, *SOC* soil organic carbon, *TKN* total kjeldahl nitrogen, *Ava* available, *EC* electrical conductivity, *Exc* exchangeable.

The values in each column followed by a different letter are significantly different according to HSD test or Friedman test ©.

*Significant at p ≤ 0.05; **significant at p ≤ 0.01; ***significant at p ≤ 0.001, *ns* not significant.

The main chemical characteristics of soils from Locone Lake are reported in Table 2. The reforested soil had significantly higher SOC (p = 0.012) and TKN (p = 0.006) content than pasture, while all other parameters did not show any significant differences.

**Table 2:** Chemical properties (mean ± standard deviation of three replicates) of Locone Lake soils.

	SOC, g kg^−1^	TKN, g kg^−1^	P_ava_, mg kg^−1^	pH (H_2_O)	pH (KCl)	EC, μS cm^−1^	Ca_exc_, g kg^−1^	K_exc_, g kg^−1^	Fe_ava_, g kg^−1^	Zn_ava_, mg kg^−1^	Cu_ava_, mg kg^−1^	Mn_ava_, mg kg^−1^	Mo_ava_, mg kg^−1^
COM	14.39 ± 1.4 (b)	1.28 ± 0.1 (b)	14.53 ± 0.7	8.02 ± 0.1	7.48 ± 0.1	239 ± 30	19.0 ± 4.9	1.93 ± 0.2	1.04 ± 0.1	27.55 ± 3.3	13.21 ± 4.9	431 ± 37.4	0.16 ± 0.7
ROM	41.13 ± 10.7 (a)	3.36 ± 0.7 (a)	17.63 ± 7.8	7.88 ± 0.2	7.25 ± 0.05	329 ± 64	19.6 ± 0.6	1.84 ± 0.1	1.03 ± 0.05	30.90 ± 2.9	16.09 ± 4.5	426 ± 51.4	0.15 ± 0.7
	*	**	ns	ns	ns	ns	ns	ns	ns	ns	ns	ns	ns

*COM* control soil; *ROM* *Robinia* *pseaudoacacia* L. soil, *SOC* soil organic carbon, *TKN* total kjeldahl nitrogen, *Ava* available, *EC* electrical conductivity, *Exc* exchangeable.

The values in each column followed by a different letter are significantly different according to HSD test.

*Significant at p ≤ 0.05; **significant at p ≤ 0.01; *ns* not significant.

Table 3 reports the microbial and enzymatic properties of Lama Giulia soils. The cypress soil showed significantly higher MBC (p = 0.0001) and DOC (p = 0.003) content than black locust and control soil. In addition, also the MBC/SOC ratio was significantly higher (p = 0.011) in the cypress soil than the other two ones. Finally, the protease activity was significantly higher (p = 0.02) in reforested soils than pasture.

**Table 3:** Enzymatic activities, microbial biomass C and dissolved organic C of Lama Giulia soils (mean ± standard deviation of three replicates).

	Dehydrogenase, mg INTF g^−1^ h^−1^	Phosphatase, µmol PNP g^−1^ h^−1^	β-glucosidase, µmol PNG g^−1^ h^−1^	Protease, µmol NH_4_^+^ g^−1^ h^−1^		Urease, µmol NH_4_^+^ g^−1^ h^−1^	MBC, mg kg^−1^	DOC, mg kg^−1^	Sol. carb, mg kg^−1^	MBC/SOC, %	Phenolic compounds, mg *p*-coumaric acid kg^−1^
COG	3.86 ± 1.20	528.2 ± 116	120 ± 78	0.28 ± 0.05 (b)		0.34 ± 0.1	281.91 ± 84 (b)	582.94 ± 123 (b)	272.3 ± 42	0.60 ± 0.3 (b)	27.95 ± 12
CYG	4.20 ± 0.4	672.6 ± 162	255.5 ± 89	0.55 ± 0.1 (a)		0.56 ± 0.2	1295.68 ± 101 (a)	1029.56 ± 120 (a)	368.5 ± 76	1.37 ± 0.1 (a)	64.81 ± 23.5
ROG	3.05 ± 0.05	510.1 ± 133	169.7 ± 59	0.55 ± 0.1 (a)		0.47 ± 0.03	497.34 ± 84 (b)	628.91 ± 61 (b)	238 ± 42	0.87 ± 0.2 (ab)	41.93 ± 7.0
	ns	ns	ns	*		ns (C)	***	*	ns	*	ns

*COG* control soil, *CYG* cypress soil, *ROG* *Robinia* *pseudoacacia* L. Soil, *MBC* microbial biomass carbon, *DOC* dissolved organic carbon, *Sol.* *carb.* soluble carbohydrates, *SOC* soil organic carbon.

The values in each column followed by a different letter are significantly different according to HSD test or Friedman test (C).

*Significant at p ≤ 0.05; ***significant at p ≤ 0.001; *ns* not significant.

The microbial and enzymatic characteristics of Locone Lake soils are reported in Table 4. The soil from black locust showed significantly higher phosphatase (p = 0.016), protease (p = 0.048) and urease (p = 0.03) activities than pasture, together to significantly higher DOC (p = 0.0074) and phenolic compounds (p = 0.043) content. In contrast, the MBC/SOC ratio was significantly lower (p = 0.0061) in reforested soil than pasture.

**Table 4:** Enzymatic activities, microbial biomass C and dissolved organic C of Locone Lake soils (mean ± standard deviation of three replicates).

	Dehydrogenase, mg INTF g^−1^ h^−1^	Phosphatase, µmol PNP g^−1^ h^−1^	β-glucosidase, µmol PNG g^−1^ h^−1^	Protease, µmol NH_4_^+^ g^−1^ h^−1^	Urease, µmol NH_4_^+^ g^−1^ h^−1^	MBC, mg kg^−1^	DOC, mg kg^−1^	Sol carb, mg kg^−1^	MBC/SOC, %	Phenolic compounds, mg *p*-coumaric acid kg^−1^
COM	4.07 ± 0.5	141.63 ± 38.4 (b)	169.00 ± 76.9	0.31 ± 0.2 (b)	0.28 ± 0.1 (b)	365 ± 6.5	485 ± 34 (b)	131 ± 22	2.57 ± 0.4 (a)	18.5 ± 3.3 (b)
ROM	4.20 ± 0.2	380.67 ± 96.3 (a)	171.68 ± 94.3	1.04 ± 0.4 (a)	0.66 ± 0.2 (a)	416 ± 138	624 ± 34 (a)	239 ± 68	1.04 ± 0.3 (b)	37.5 ± 11 (a)
	ns	*	ns	*	*	ns	**	ns	**	*

*COM* control soil, *ROM* *Robinia* *pseudoacacia* L. soil, *MBC* microbial biomass carbon, *DOC* dissolved organic carbon, *Sol.* *carb.* soluble carbohydrates, *SOC* soil organic carbon.

The values in each column followed by a different letter are significantly different according to HSD test.

*Significant at p ≤ 0.05; **significant at p ≤ 0.01; *ns* not significant.

**Figure 3:**
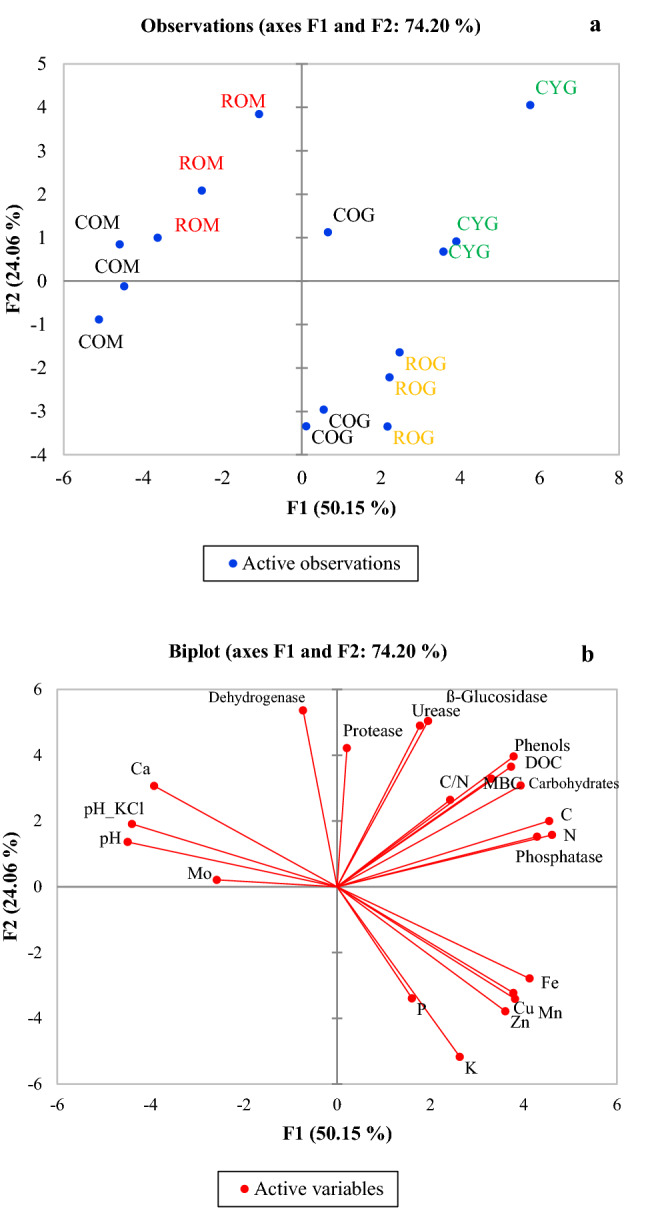
Principal component analysis shown as biplots of scores. (**a**) Sites, (**b**) variables loadings. *ROM* black locust at Locone Lake, *COM* degraded but not reforested soils at Locone Lake, *CYG* cypress at Lama Giulia, *ROG* black locust at Lama Giulia, *COG* degrade but not reforested soils at Lama Giulia.

The PCA analysis isolated five principal components, but only the first two were considered because they explained 74.2% of the variability (Fig. 3). Along the first principal component F1 there was a clear segregation between the points belonging to Lama Giulia (on the right) and those belonging to Locone Lake (on the left) (Fig. 3a). This result explained 50.15% of variability and it was mainly supported by SOC (load 0.940), TKN (load 0.952), soluble carbohydrates (load 0.813), phenolic compounds (load 0.783) and DOC (load 0.771) content, pH value (load −0.928 and −0.909 for pH_H2O_ and pH_KCl_, respectively) and phosphatase activity (load 0.886) and, to a lesser extent, by Ca, Fe, Zn, Cu and Mn content (Fig. 3b; Table 5). Along the second principal component F2, the black locus from Lama Giulia (ROG) was clearly segregated from the black locust of the other site (ROM) and from the cypress of the same site (CYG) (Fig. 3a). This result explained 20.06% of variability and it was mainly supported by K content (load −0.740), and by glucosidase (load 0.722), urease (load 0.701) and dehydrogenase (load 0.768) activities (Table 5).

**Table 5:** PCA loadings.

Parameters	F1	F2
Soil organic carbon (SOC)	0.940	0.287
Total kjeldahl nitrogen (TKN)	0.952	0.226
C/N	0.501	0.378
P (available)	0.332	−0.485
pH (H2O)	−0.928	0.195
pH (KCl)	−0.909	0.273
Ca (exchangeable)	−0.811	0.439
K (exchangeable)	0.544	−0.740
Fe (available)	0.853	−0.399
Zn (available)	0.746	−0.542
Cu (available)	0.788	−0.487
Mn (available)	0.781	−0.462
Mo (available)	−0.533	0.030
Phosphatase activity	0.886	0.218
Glucosidase activity	0.403	0.722
Urease activity	0.367	0.701
Protease activity	0.043	0.604
Dehydrogenase activity	−0.151	0.768
Microbial biomass carbon (MBC)	0.684	0.470
Dissolved organic carbon (DOC)	0.771	0.523
Soluble carbohyidrates	0.813	0.440
Phenolic compounds	0.783	0.567

## Discussion

The two sites were segregated for their SOC content, with Lama Giulia soils showing a general higher SOC content than Locone Lake site. The larger SOC content of the cypress soil than black locust soils could be the result of about forty years of litter deposition occurred in the former soil with respect to the about thirty and seventeen years of black locust litter deposition at Locone Lake and Lama Giulia sites, respectively. In addition, the quality of the plant residues could have also played a role in C sequestration since the litter contribution is related to the type of plant^28^ and, in particular, black locust litter has a relatively higher input of aliphatic carbon, while the conifer litter composition is more aromatic and shows a slower decomposition^29^.

The Lama Giulia site differed from Locone Lake one for the TKN content too. Although a higher TKN content was expected in black locust soils because of the biological N fixation (BNF), the concentration of TKN was the highest in the cypress soil and mainly related to the litter deposition over time, since 90–95% of soil N is organic. Certainly, cypress also establishes relationships with beneficial microorganisms of the rhizosphere, while the BNF can be influenced by environmental conditions. Wei et al.^30^ reported that *R.* *pseudoacacia* L. forms nodules with many different bacterial species and this can explain the environmental success of black locust, but Schulze et al.^31^ and Zahran^32^ reported that the symbiotic association and the BNF are sensitive to soil water availability and drought stress. For example, Veste and Kriebitzsch^33^ demonstrated that, under long-term drought stress, the leaf N content of black locust reduced due to the negative influence of extreme drought on BNF. Even though *Robinia* *pseudoacacia* L. can grow in semi-arid environments, such as the Apulia region ones, it originated from climatic regions with annual rainfall of 1020 to 1830 mm^34^. Therefore, since in Apulia the balance between rainfall and evapotranspiration is frequently negative during late spring and summer, the drought may have affected the BNF.

The pH values of soils from Lama Giulia were lower than those from Locone Lake and inversely proportional to their SOC content since the soil organic matter offers a large source of acidic protons in the form of carboxyl groups that can determine a decrease of pH values^35^.

According to the PCA analysis, Locone Lake site showed a general lower phosphatase activity than Lama Giulia and, within each site, no significant difference was found in Lama Giulia (Table 3), while the black locust of Locone Lake showed significantly larger phosphatase activity than the corresponding pasture (Table 4). Since the phosphatase activity is mostly promoted by higher content of soil organic P and total N, and lower pH, the results are in accordance with the trend of those variables between the two sites and within each site. However, P_ava_ has been demonstrated that did not correlate with phosphatase activity^36^. The highest content of P_ava_ recorded in ROG almost certainly was related to the grazing cattle present in that area which enriched the site with their excreta, since black locust is also a food source for livestock and wild herbivores. This result is apparently confirmed also by the significantly higher content of Zn and Cu in the black locust soil within the Lama Giulia site (ROG), since those elements are often related to the animal droppings.

The two sites were segregated also by the soluble carbohydrates, phenolic compounds and DOC that represent labile fractions of the soil organic matter directly affected by the aboveground and belowground litter inputs. In general, the significantly largest DOC content and the numerically higher carbohydrates and phenolic compounds contents of the cypress soil suggest that the quality of the cypress litter has a better effect than black locust and pastures litters to maintain a pool of labile organic C that provide a source of energy for soil microbial activities. In fact, the chemical composition of the litter species such as lignin, polyphenols, and the C-to-N and lignin-to-N ratios control the litter decomposition and the environmental conditions for soil microbiota^37^. Indeed, the majority of enzymatic activities are correlated with SOC and the easily degradable organic compounds in soil^38,39^, which stimulate the growth of soil microbial community.

The PCA analysis indicated, even to a lesser extent, that CYG and ROM soils shared a slightly higher dehydrogenase activity than ROG. Dehydrogenase activity is an index of the global soil microbial metabolic activity and health status of the soil ecosystem^40^. Dehydrogenase is exclusively an intracellular enzyme that is only present in living microbial cells and therefore is a well-founded marker for monitoring changes in soil metabolism^41^. As the ecosystems develop with time, there is a reduction of metabolic activity and tendency to reach energy equilibrium in the system^42,43^, therefore the results pointed out that all soils showed different environmental conditions that modulated the changes in the overall soil metabolism. This result was confirmed also by the activities of the extracellular enzymes β-glucosidase and urease, involved in organic matter turnover and in soil N metabolism, respectively^44^. In fact, they showed lower activities in ROG soil than the other tree soils.

The protease showed a significantly tree-related stimulation of its activity over the pasture soils within each site (Tables 3 and 4), in response to the higher TKN content of such soils than corresponding pastures that stimulated the synthesis of this enzyme by soil microbial community^45^.

The MBC is a reliable indicator of changes in soil quality reflecting the long-term effects of soil-vegetation system and it was positively correlated to the SOC contents (R^2^ = 0.84). Even if the MBC did not segregate the two sites, within the Lama Giulia one it reached the significantly highest value in the cypress soil because of the longer inputs of litter that increased the food resources for microbes and the amount of soil microorganisms. In addition, De Marco et al.^46^ found that black locust soils had low MBC and respiratory response because of the presence of inhibitory substances, such as 4-hydroxyacetophenon, in black locust litter extracts, that negatively affects the abundance and activity of the soil microbial community and the microbial-derived organic matter formation.

Soil microbial entropy, defined as the ratio of soil MBC to SOC, is a sensitive indicator of the soil quality, assessing the SOC quality and its turnover rate^47^. It reflects the percentage of the active SOC in the soil ecosystem and the changes of soil environment^48^. MBC generally account for 1–4% of the total SOC^49^. Comparing the two pastures, the higher soil microbial entropy of COM with respect to COG was possibly due to the sandy texture of the former soil, that ensured a higher SOC turnover. For the same reason, ROM had higher MBC/SOC ratio than ROG. Within Lama Giulia site, CYG showed the slightly but significantly highest soil microbial entropy because of the amount of litter deposition and, to a lesser extent, the quality of the litter.

## Conclusions

The effects of *Robinia* *pseudocacia* L. on Locone Lake were remarkable, with an increase of SOC and TKN content in comparison to the corresponding pasture. Comparing the two black locust plantations, the different soil texture between the two sites determined higher values of such parameters in Lama Giulia than Locone Lake, despite the latter was reforested earlier than the former site. In fact, the Locone Lake’s sandy soils showed a higher microbial entropy and turnover, especially in the not reforested soil, suggesting a less conservative soil state than Lama Giulia’s silty loam soil.

Within the Lama Giulia site, CYG soil showed the highest values of some chemical and microbial parameters due to the longest time of reforestation. In contrast, almost all soil parameters studied were not statistically different between the ROG and the corresponding pasture, suggesting that about 17 years of reforestation with black locust apparently are not sufficient to establish general better soil quality.

## Data Availability

The data presented in this study are available within the article.
